# Reference intervals for intact FGF 23 in healthy Korean adults: lower concentrations in young adulthood require age-specific partitioning

**DOI:** 10.3389/fendo.2026.1730871

**Published:** 2026-03-06

**Authors:** Yonggeun Cho, Hanmil Jang, Hyun-June Nam, Jaehyeok Jang, Hyein Kang, John Hoon Rim, Sang-Guk Lee, Jong-Baeck Lim

**Affiliations:** 1 Department of Laboratory Medicine, Hallym University Sacred Heart Hospital, Anyang, Republic of Korea; 2 Department of Laboratory Medicine, Severance Hospital, Yonsei University College of Medicine, Seoul, Republic of Korea; 3 Department of Biosciences, Yonsei University College of Medicine, Seoul, Republic of Korea; 4 Department of Laboratory Medicine, Keimyung University School of Medicine, Daegu, Republic of Korea

**Keywords:** age partitioning, chemiluminescent immunoassay, fibroblast growth factor 23, Korean population, mineral metabolism, reference interval

## Abstract

**Background:**

Fibroblast growth factor 23 (FGF23) is a bone-derived phosphaturic hormone that is essential for phosphate homeostasis. Elevated FGF23 levels underlie FGF23-related hypophosphatemic rickets and tumor-induced osteomalacia. Despite its clinical importance, population-based reference intervals (RIs) for intact FGF23 using the widely deployed LIAISON XL automated chemiluminescent immunoassay platform (DiaSorin) are lacking for East Asian populations.

**Methods:**

We established method-specific RIs for intact FGF23 (iFGF23) in 386 healthy Korean adults (193 males and 193 females; age, 20–79 years) following the Clinical and Laboratory Standards Institute EP28-A3c guidelines. After the Box–Cox transformation and Horn’s outlier detection, the RIs were derived using nonparametric methods (2.5th–97.5th percentiles). The necessity for partitioning was assessed using the Harris–Boyd method. Associations between iFGF23 levels and demographic, anthropometric, and biochemical parameters were examined using Pearson’s correlation coefficients.

**Results:**

The overall nonparametric RI was 28.04–100.33 pg/mL (90% CI: 25.77–31.91 to 96.29–109.20). Age emerged as the primary determinant requiring partitioning, with young adults (20–29 years) exhibiting significantly lower concentrations than older adults (≥30 years): 25.73–78.76 versus 32.01–107.00 pg/mL. A sex-stratified analysis confirmed that this age effect persisted independently in both males and females. Although males had higher median iFGF23 than females (65.03 vs. 51.98 pg/mL, *p* < 0.001), Harris–Boyd analysis did not support sex-based partitioning (*z* = 5.17, z* = 5.38). Intact FGF23 was significantly correlated with age (*r* = 0.278), estimated glomerular filtration rate (*r* = –0.254), and alkaline phosphatase (*r* = 0.143; all *p* ≤ 0.005), but not with traditional mineral metabolism parameters (phosphate, calcium, parathyroid hormone, and 25-hydroxyvitamin D).

**Conclusions:**

This study provides the first population- and method-specific RIs for intact FGF23 in an East Asian population and establishes critical age-stratified benchmarks for clinical interpretation. The distinct RI in young adults underscores the necessity of age-appropriate reference standards for diagnosing and monitoring phosphate homeostasis disorders. These findings highlight the importance of population-specific reference data in the absence of assay harmonization.

## 1 Introduction

Fibroblast growth factor 23 (FGF23) is a bone-derived phosphaturic hormone that plays a key role in maintaining phosphate homeostasis (1). Secreted in response to hyperphosphatemia, FGF23 reduces serum phosphate concentrations through two principal mechanisms: inhibition of renal tubular phosphate reabsorption and suppression of 1,25-dihydroxyvitamin D_3_ (calcitriol) synthesis (2). The pathological elevation of FGF23 levels underlies a spectrum of renal phosphate-wasting disorders, collectively designated as FGF23-related hypophosphatemic rickets (FGF23rHR). This disease entity encompasses inherited forms, including X-linked hypophosphatemia, autosomal dominant hypophosphatemic rickets, and autosomal recessive hypophosphatemic rickets, as well as the acquired paraneoplastic syndrome tumor-induced osteomalacia (3).

Quantification of circulating FGF23 concentrations has become increasingly important in the diagnostic evaluation of these disorders, with thresholds established from chronic hypophosphatemic patient cohorts (4–7). Although these decision limits are clinically useful for differentiating disease states, they do not fulfill the distinct methodological requirements of population-based reference intervals (RIs). According to the International Federation of Clinical Chemistry and Laboratory Medicine, RIs are defined as the expected distribution of analyte values in healthy reference populations and constitute an essential prerequisite for valid interpretation of individual laboratory measurements (8). Proper interpretation of FGF23 measurements, whether for assessing mineral homeostasis alongside phosphate and calcium (9) or for therapeutic monitoring with the recently approved anti-FGF23 monoclonal antibody burosumab (10), requires establishment of robust RIs.

The bioactivity of FGF23 is stringently regulated through proteolytic cleavage at a conserved furin-sensitive motif (RXXR), which generates biologically inert N- and C-terminal fragments (11); only the intact, full-length molecule retains hormonal activity. Consequently, two distinct immunoassay architectures have been developed: C-terminal assays, which detect both intact hormones and cleavage fragments, and intact assays, which employ two-site sandwich configurations that selectively quantify the bioactive intact form (1). Critically, no higher-order reference measurement procedure or certified reference material has been established for FGF23 (1), and comparative studies using patient samples have consistently documented substantial intermethod bias (4, 6, 12–14).

The LIAISON FGF23 assay (Diasorin S.p.A., Saluggia, Italy) is a widely implemented, fully automated, two-site chemiluminescent immunoassay that quantifies intact FGF23. Although population-based RIs for this platform have been reported in a large French cohort (12), comparable data from East Asian populations are lacking. Published Japanese reference data (5–7, 15) have predominantly employed alternative methodologies, including manual enzyme-linked immunosorbent assays (ELISA) and distinct chemiluminescent enzyme immunoassay (CLEIA) platforms, which yield systematically divergent concentrations that preclude direct comparability. In the absence of assay standardization and considering potential interethnic physiological variations, the establishment of a method- and population-specific RI is clinically essential. This study addressed that unmet need by establishing robust RIs for intact FGF23 (iFGF23) in healthy Korean adults using the LIAISON XL platform (DiaSorin).

## 2 Materials and methods

### 2.1 Study population and sample collection

This study was approved by the Institutional Review Board of Severance Hospital (approval number 1-2022-0019). Informed consent was waived for the use of remnant specimens. Remnant K_3_EDTA plasma samples were obtained from healthy adults undergoing preventive health assessment at the Severance Health Check-up Center between December 2022 and November 2023. All samples were collected after an overnight fast on the morning of the visit. Eligibility required fulfillment of strict biochemical criteria: serum phosphate 2.5–4.5 mg/dL, serum calcium 8.5–10.5 mg/dL, and estimated glomerular filtration rate (eGFR) ≥60 mL/min/1.73 m^2^. The exclusion criteria were pregnancy, known hypophosphatemic rickets, chronic kidney disease, metabolic bone disease, active malignancy, infectious disease, or autoimmune disorders.

### 2.2 Anthropometric measurements

Height, weight, body fat mass, and skeletal muscle mass were determined using bioelectrical impedance analysis (InBody, InBody Co., Ltd., Seoul, Korea).

### 2.3 Biochemical analyses

Routine serum biochemistry including phosphate, calcium, and creatinine levels, was performed using an automated clinical chemistry analyzer (Cobas c702, Roche Diagnostics, Mannheim, Germany). The eGFR was calculated from serum creatinine using the 2009 CKD-EPI equation (16), rather than the 2021 race-free version (17), due to the latter’s lower reported accuracy in Korean populations (18). Intact parathyroid hormone (PTH) and 25-hydroxyvitamin D levels were measured using a chemiluminescent immunoassay analyzer (Cobas e801, Roche Diagnostics); the internal quality control CVs during the study period were 2.0-2.9% for PTH and 4.4-5.3% for 25-hydroxyvitamin D. Plasma concentrations of iFGF23 were quantified using a two-site chemiluminescent immunoassay on the LIAISON XL platform.

### 2.4 Reference interval derivation

#### 2.4.1 Outlier detection

To ensure robustness, we applied the Horn algorithm for outlier screening (19). Briefly, measurements were first transformed using the Box-Cox procedure to approximate normality, after which Tukey interquartile fences were computed. Values more than 1.5 × the interquartile range below the first quartile or above the third quartile were flagged and excluded before RI estimation.

#### 2.4.2 Reference interval estimation

The RIs were established in accordance with the Clinical and Laboratory Standards Institute (CLSI) EP28-A3c guidelines. The nonparametric percentile method (2.5th–97.5th percentiles) served as the primary approach, because it required no distributional assumptions. Parametric methods were additionally applied to log transformed and Box–Cox transformed data for confirmatory analyses. Robust estimations were also obtained using Horn’s method. Normality was assessed using the Shapiro–Wilk test, Anderson–Darling test, and visual inspection of the quantile–quantile plots.

#### 2.4.3 Subgroup partitioning

The necessity for subgroup-specific RIs was evaluated using the Harris–Boyd method (20), with modifications to accommodate comparisons involving more than two subgroups. First, iFGF23 values were Box–Cox transformed to an approximate normal distribution as required for parametric partitioning. Second, subgroups were compared using Student’s *t*-test (for two groups) or one-way ANOVA with Tukey’s Honestly Significant Difference (HSD) as the *post-hoc* test (for multiple groups) to identify statistically different means. Third, the Harris–Boyd z-statistic was calculated by Equation 1 for each significant pairwise comparison.

(1) $$z\;=\;\frac{|{\bar{x}}_{1}-\;{\bar{x}}_{2}|}{{\left(\sigma_{1}^{2}/n_{1}+\sigma_{2}^{2}/n_{2}\right)}^{1/2}}$$

where ${\bar{x}}_{1}$ and ${\bar{x}}_{2}$ are subgroup means, σ_1_^2^ and σ_2_^2^ are variances, and n_1_ and n_2_ are sample sizes.

The critical value z* is calculated by Equation 2 to serve a s a partitioning threshold (when z > z*).

(2) $$z^{*}=3\cdot {\left((n_{1}+n_{2})/120\;\right)}^{1/2}$$

Fourth, when partitioning was warranted, subgroup-specific RIs were derived from untransformed iFGF23 values using a nonparametric method (2.5th–97.5th percentiles). Candidate partitions included sex, age groups, body mass index (BMI) (<18.5, 18.5–24.9, 25–29.9, ≥30 kg/m^2^), and eGFR (60–89, ≥90 mL/min/1.73 m^2^).

### 2.5 Correlation analyses

Associations between iFGF23 and demographic, anthropometric, and biochemical variables were examined using Pearson’s correlation coefficients. Covariates included age, eGFR, and serum phosphate, calcium, PTH, 25-hydroxyvitamin D, and alkaline phosphatase levels. Statistical significance was defined as two-sided *p* < 0.05.

### 2.6 Age-stratified comparisons

To characterize age-related variations in biochemical profiles, participants were grouped into young adults (20–29 years) and older adults (≥30 years) based on the partitioning analysis results. Between-group differences in continuous variables were evaluated using the Mann–Whitney *U* test, and sex ratio differences were assessed using the chi-square test. Continuous variables were expressed as medians with interquartile ranges.

### 2.7 Sex-stratified age partitioning analysis

As a supplementary analysis to evaluate the robustness of age partitioning independent of sex, Box–Cox transformed iFGF23 values were compared across age groups separately for males and females. One-way ANOVA was used to assess the overall age effects within each sex, followed by Tukey’s HSD *post-hoc* test for pairwise comparisons, with the 20–29 year age group serving as the reference. Statistical significance was defined as *p* < 0.05.

### 2.8 Statistical software

All analyses were conducted using the R software (version 4.3.1; R Foundation for Statistical Computing, Vienna, Austria). The referenceIntervals package (version 1.2.0) facilitated the RI establishment per CLSI EP28-A3c specifications, incorporating Horn’s outlier detection. Box–Cox transformations were performed using the car package (version 3.1-2). Data management was performed using Microsoft Excel 2016 and graphics were generated using ggplot2 package (version 3.4.2).

## 3 Results

### 3.1 Distribution characteristics and outlier exclusion

**Figure 1:**
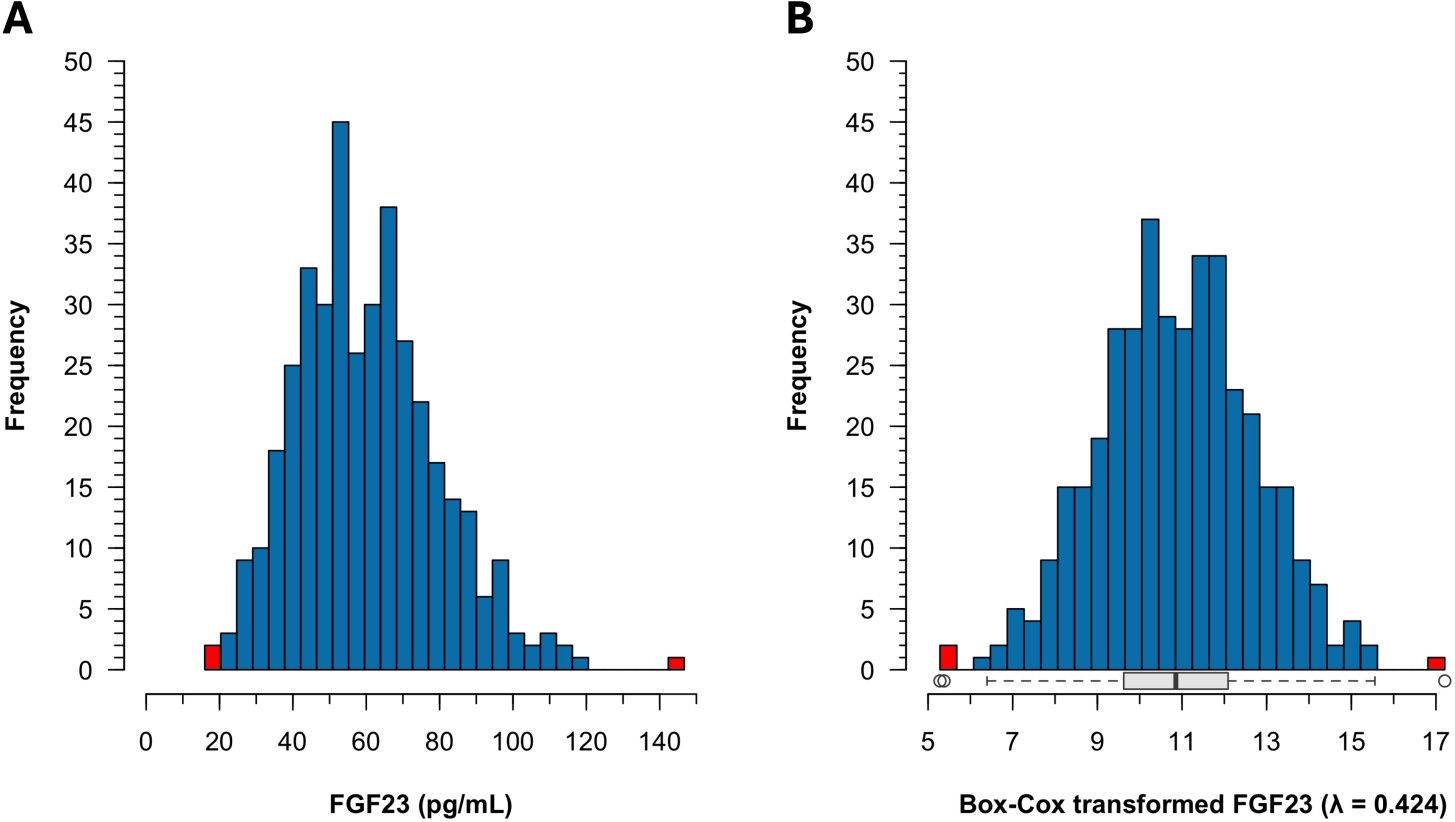
Distribution of plasma intact FGF23 concentrations in 389 healthy Korean adults. **(A)** Histogram showing right-skewed distribution. **(B)** Histogram after Box–Cox transformation (λ = 0.424) with box-and-whisker plot below. Three outliers (highlighted in red) were identified using Horn’s robust method based on Box–Cox transformed values.

A total of 389 remnant plasma samples (194 male, 195 female) were initially evaluated. On the original scale, plasma iFGF23 exhibited moderate positive skewness (0.594) and excess kurtosis (0.663), with a significant departure from normality (Shapiro–Wilk *W* = 0.982, *p* < 0.001; Anderson–Darling *A* = 1.539, *p* < 0.001; **Figure 1A**). Box–Cox transformation of the complete dataset identified an optimal power parameter λ = 0.424, yielding transformed values that satisfied normality criteria (Shapiro–Wilk *W* = 0.999, *p* = 0.995; Anderson–Darling *A* = 0.131, *p* = 0.982**;****Figure 1B**). Application of Horn’s algorithm to these transformed data identified three statistical outliers with original-scale concentrations of 15.98 pg/mL, 16.46 pg/mL, and 146.70 pg/mL. Following the exclusion of these individuals (two female and one male), the final reference population comprised 386 participants (193 male and 193 female). Given the removal of extreme values, Box–Cox transformation was re-applied to the cleaned dataset, yielding a revised optimal parameter λ = 0.343 with retained normality (Shapiro–Wilk *W* = 0.997, *p* = 0.663; Anderson–Darling *A* = 0.203, *p* = 0.876). This reference population was used for all subsequent RI derivation and partitioning analyses.

### 3.2 Baseline characteristics of reference population

The baseline demographic and biochemical characteristics, stratified by sex, are presented in **Supplementary Table 1**. The final reference cohort comprised 193 males and 193 females with a median age of 53 years (IQR, 28–63 years). Males demonstrated significantly higher BMI (24.7 vs. 22.1 kg/m^2^, *p* < 0.001), skeletal muscle mass (29.8 vs. 20.5 kg, *p* < 0.001), and median iFGF23 (65.03 vs. 51.98 pg/mL, *p* < 0.001) compared with females. Conversely, females exhibited higher serum phosphate (3.9 vs. 3.5 mg/dL, *p* < 0.001) and marginally lower calcium (9.5 vs. 9.6 mg/dL, *p* = 0.003). PTH and 25-hydroxyvitamin D did not differ significantly between sexes.

### 3.3 Establishment of primary reference interval

RIs were derived using multiple statistical approaches (**Table 1**). In the reference population (*n* = 386), the nonparametric method (2.5th–97.5th percentiles) yielded 28.04–100.33 pg/mL (90% CI: 25.77–31.91 to 96.29–109.20). Parametric methods produced comparable estimates: Box–Cox transformed data yielded 28.97–102.07 pg/mL (90% CI: 27.33–30.67 to 98.31–105.93), while log transformed data yielded 30.47–107.27 pg/mL (90% CI: 29.10–31.90 to 102.46–112.32). The assumption of normality for the log transformed data was equivocal, with the Shapiro-Wilk test suggesting non-normality (*p* = 0.023) while the Anderson-Darling test did not (*p* = 0.065). Robust estimation produced a narrower interval of 21.63–95.53 pg/mL (90% CI: 18.85–24.18 to 92.48–98.39). According to CLSI guidelines (21), the distribution-free nonparametric method was designated as the primary RI.

**Table 1:** Reference intervals of intact FGF23 (pg/mL) in reference population (*n* = 386).

Method	Group	*n*	2.5th percentile [90% CI]		97.5th percentile [90% CI]	
Non-parametric	Total	386	28.04	[25.77–31.91]	100.33	[96.29 – 109.20]
	Age					
	20–29 years	124	25.73	[22.01–28.31]	78.76	[74.98–88.62]
	≥30 years	262	32.01	[26.58–33.89]	107.00	[98.21–113.50]
Parametric						
Log transformation^a^	Total	386	30.47	[29.10 – 31.90]	107.27	[102.46 – 112.32]
Box–Cox transformation^b^	Total	386	28.97	[27.33 – 30.67]	102.07	[98.31 – 105.93]
Robust	Total	386	21.63	[18.85 – 24.18]	95.53	[92.48 – 98.39]

a Following log transformation, the Shapiro-Wilk test indicated a significant deviation from normality (*W* = 0.991, *p* = 0.023), whereas the Anderson-Darling test did not (*A* = 0.705, *p* = 0.065).

b The Box–Cox transformation successfully normalized the data, as confirmed by non-significant results from both the Shapiro-Wilk (*W* = 0.997, *p* = 0.663) and Anderson-Darling (*A* = 0.203, *p* = 0.876) tests.

### 3.4 Partitioning analysis and establishment of subgroup reference intervals

**Figure 2:**
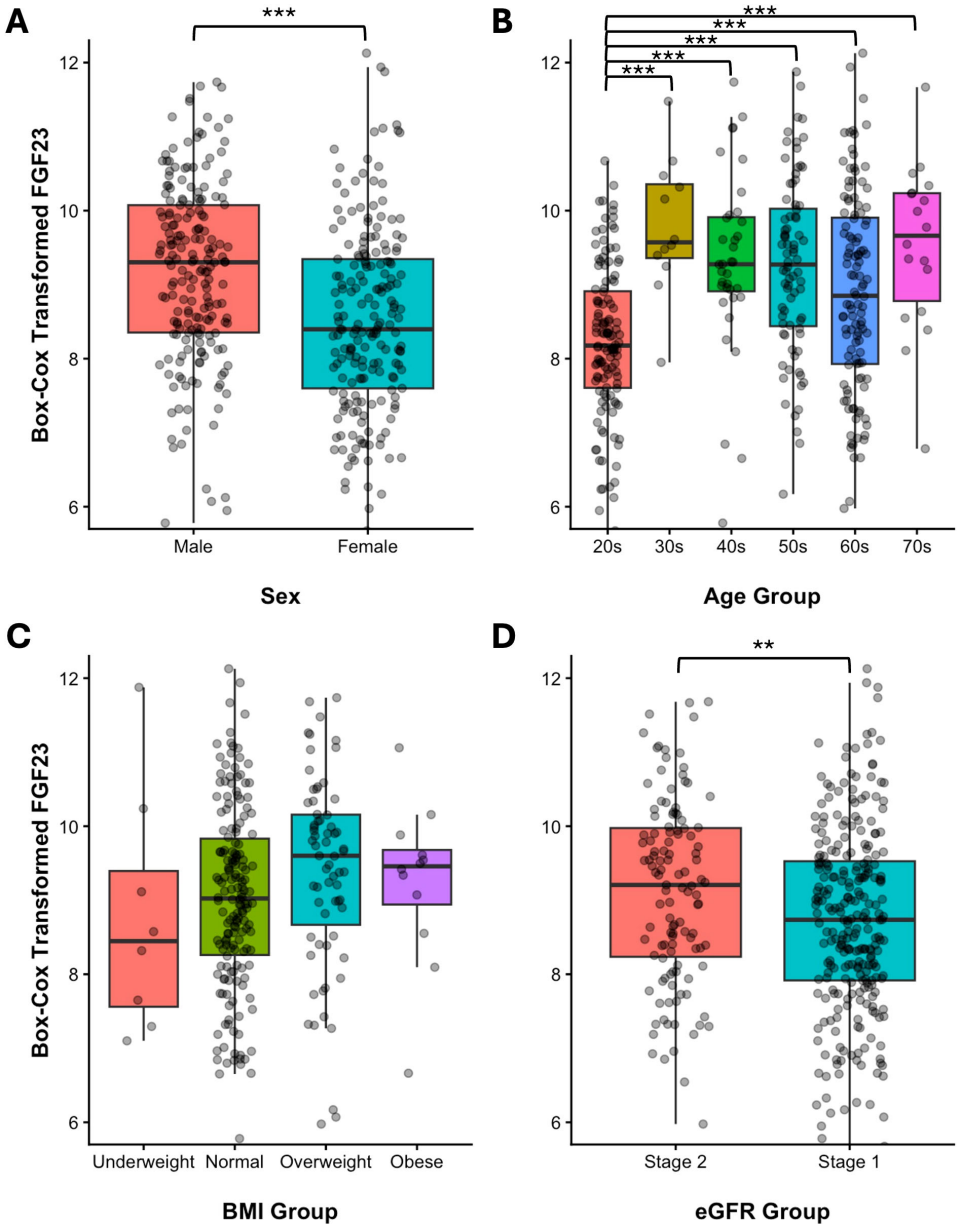
Distribution of intact FGF23 concentrations across subgroups. Dot plots and boxplots of Box–Cox transformed (λ = 0.343) intact FGF23 values stratified by **(A)** sex, **(B)** age group, **(C)** BMI categories (underweight <18.5, normal 18.5–24.9, overweight 25.0–29.9, obese ≥30 kg/m^2^), and **(D)** eGFR categories (Stage 2: 60–89 mL/min/1.73 m^2^; Stage 1: ≥90 mL/min/1.73 m^2^). Statistical significance determined by Student’s *t*-test for binary comparisons and one-way ANOVA with Tukey’s HSD *post-hoc* test for multiple comparisons: *p < 0.05, **p < 0.01, ***p < 0.001.

Partitioning necessity was assessed using the Harris–Boyd method on Box–Cox transformed iFGF23 values (λ = 0.343) to satisfy normality requirements (**Table 2**, **Figure 2**).

**Table 2:** Partitioning assessment of intact FGF23 using box–cox transformed values (λ = 0.343).

Variable	*n*	mean	SD	*p*-value^a^	*Post-hoc* ^b^	z	z*
Sex				< 0.001		5.17	5.38
male	193	9.17	1.21				
female	193	8.52	1.26				
Age				< 0.001			
20–29	124	8.22	1.05		all others (≥30)		
30–39	12	9.78	0.91		20–29	5.59^c^	3.19
40–49	36	9.31	1.23		20–29	4.83^c^	3.46
50–59	80	9.25	1.20		20–29	6.28^c^	3.91
60–69	116	8.89	1.34		20–29	4.29^c^	4.24
70–79	18	9.52	1.13		20–29	4.60^c^	3.26
BMI				0.213			
	8	8.77	1.62				
18.5–24.9	170	9.03	1.24				
25–29.9	67	9.39	1.31				
≥30	12	9.24	1.10				
eGFR				0.005		2.90	5.38
60–89	119	9.12	1.24				
≥90	267	8.72	1.28				

Age in years, BMI in kg/m^2^, and eGFR in mL/min/1.73 m^2.^

a One-way ANOVA for factors with ≥3 categories; two-sample Student’s *t*-test for binary factors.

b Groups showing statistically significant differences from each subgroup in Tukey’s HSD *post-hoc* test (p < 0.05) following significant ANOVA results.

c Indicates partitioning is recommended by the Harris–Boyd method (where z > z*).

SD, standard deviation; BMI, body mass index; eGFR, estimated glomerular filtration rate.

**Sex:** Although males exhibited a significantly higher mean iFGF23 level than females (*p* < 0.001), the z-statistic (z = 5.17) fell slightly below the critical threshold (z* = 5.38), indicating that partitioning was not statistically warranted. Supporting this decision, the 90% CIs of the sex-specific nonparametric reference limits showed substantial overlap (males: 25.54–36.46 to 96.19–109.20 pg/mL; females: 23.31–31.63 to 89.07–114.90 pg/mL), suggesting that separate intervals would not provide clinically meaningful improvement in diagnostic accuracy.

Age: A highly significant age effect was observed (*p* < 0.001). *Post-hoc* analysis revealed that individuals aged 20–29 years exhibited substantially lower iFGF23 levels than all older age strata, with z-statistics substantially exceeding the critical thresholds (z = 4.29–6.28, all z* ≤ 4.24). Accordingly, the cohort was partitioned into young adults (20–29 years) and adults (≥30 years), yielding distinct RIs of 25.73–78.76 and 32.01–107.00 pg/mL, respectively (**Table 1**).

Other variables: Although the eGFR strata differed significantly (*p* = 0.005), the z-statistic (z = 2.90) was well below the critical threshold (z* = 5.38), obviating eGFR-based partitioning. BMI categories showed no significant differences (*p* = 0.213).

### 3.5 Correlates of iFGF23

Pearson’s correlation analysis identified significant positive associations between iFGF23 and age (*r* = 0.278, *p* < 0.001) and alkaline phosphatase (*r* = 0.143, *p* = 0.005), as well as a significant inverse relationship with eGFR (*r* = –0.254, *p* < 0.001) (**Supplementary Table 2**, **Supplementary Figure 1**). No significant linear associations were observed between iFGF23 levels and serum phosphate, calcium, PTH, or 25-hydroxyvitamin D levels (all *p* > 0.05).

### 3.6 Age-stratified biochemical profiles

Baseline characteristics stratified by age group (20–29 vs. ≥30 years) are presented in **Table 3**. The older age group had a higher proportion of males (54.2% vs. 41.1%, *p* = 0.022). Compared with young adults, older adults exhibited significantly reduced eGFR (median 91.5 vs. 114.5 mL/min/1.73 m^2^, *p* < 0.001) and significantly elevated concentrations of iFGF23 (64.06 vs. 49.09 pg/mL, *p* < 0.001), PTH (33.1 vs. 28.65 pg/mL, *p* = 0.002), 25-hydroxyvitamin D (30.5 vs. 26.20 ng/mL, *p* < 0.001), and alkaline phosphatase (70 vs. 61 IU/L, *p* < 0.001). Conversely, serum phosphate levels were higher in young adults (3.9 vs. 3.6 mg/dL, *p* < 0.001). Serum calcium levels did not differ between age strata (*p* = 0.589).

**Table 3:** Comparison of characteristics between young adults (20–29 years) vs. older adults (≥30 years).

Variable	20–29 years (*n* = 124)	≥30 years (*n* = 262)	*p*-value^a^
Sex ratio, male/female	51/73	142/120	0.022
Age, years	26.0 [25.0–27.5]	60.0 [52.0–65.0]	< 0.001
eGFR, mL/min/1.73 m^2^	115 [103–122]	92 [82–98]	< 0.001
iFGF23, pg/mL	49.09 [42.05–59.28]	64.06 [51.14–76.65]	< 0.001
Phosphate, mg/dL	3.9 [3.6–4.2]	3.6 [3.3–3.9]	< 0.001
Calcium, mg/dL	9.5 [9.2–9.8]	9.6 [9.3–9.8]	0.589
PTH, pg/mL	28.7 [20.6–38.4]	33.1 [26.9–40.6]	0.002
25(OH)D, ng/mL	26.2 [21.4–29.8]	30.5 [24.9–38.5]	< 0.001
ALP, IU/L	61 [55–72]	70 [62–82]	< 0.001

Continuous variables are summarized as median [interquartile range].

a Group differences were tested with Mann–Whitney *U* test for continuous variables and chi-square test for sex ratio.

eGFR, estimated glomerular filtration rate; iFGF23, intact fibroblast growth factor 23; PTH, parathyroid hormone; 25(OH)D, 25-hydroxyvitamin D; ALP, alkaline phosphatase.

### 3.7 Sex-stratified age partitioning analysis

Although sex-based partitioning was not strictly required by the Harris–Boyd criteria (**Table 2**), the proximity of the z-statistic (z = 5.17) to the critical threshold (z* = 5.38), combined with an observed sex imbalance between the age groups (**Table 3**), prompted a supplementary analysis of age-related partitioning stratified by sex (**Supplementary Figure 2**). One-way ANOVA revealed significant differences in Box–Cox transformed iFGF23 across age groups in both males (*p* < 0.001) and females (*p* < 0.001). Tukey’s HSD *post-hoc* comparisons demonstrated that the 20–29 year age group differed significantly from the multiple older age groups in both sexes. In males, the youngest cohort showed significantly lower iFGF23 levels than those aged 30–39 years (*p* = 0.017), 50–59 years (*p* = 0.002), and 70–79 years (*p* = 0.050). In females, the group aged 20–29 years differed significantly from those aged 40–49 years (*p* = 0.007), 50–59 years (*p* < 0.001), and 60–69 years (*p* = 0.008). Notably, comparisons among older age groups (≥30 years) yielded no significant differences in either sex (all *p* > 0.05). These findings support the partitioning of young adults (20–29 years) from older adults (≥30 years) independent of sex, validating our primary age-stratification approach.

## 4 Discussion

This study established rigorous, method-specific RIs for iFGF23 in a large, well-characterized cohort of healthy Korean adults, using a fully automated LIAISON XL chemiluminescent immunoassay. To our knowledge, this is the first population-based RI derivation using this platform in East Asian populations. The nonparametric 95% RI for the overall cohort was 28.04–100.33 pg/mL (**Table 1**). A principal finding was the marked age dependence of iFGF23, with significantly lower concentrations in young adults (20–29 years old), necessitating age-stratified intervals. These data provide assay-specific interpretive benchmarks for Korean adults and address the critical gap in East Asian laboratory reference standards.

Our RI demonstrates good concordance with the 22.7–93.1 pg/mL interval reported by Souberbielle et al. in a large French cohort using the identical LIAISON XL platform (12) (**Table 4**). The modest upward shift in our interval may reflect interethnic physiological differences between the Korean and European populations or methodological variations in the statistical approach. We adopted a nonparametric method as the primary estimator owing to the right-skewed data distribution, a characteristic similar to that observed by Smith et al. (15). The validity of our nonparametric interval was corroborated by its close agreement with the parametric estimates derived from the Box–Cox transformed data.

**Table 4:** Comparison of reference intervals for intact FGF23 in adult populations.

Assay (platform)	Country	Year	Age range (years)	*n*	Statistical method	Reference interval (pg/mL)	Reference
LIAISON XL FGF23 (CLIA)	Korea	2025	20–79	386	Nonparametric (2.5th–97.5th)	28.04–100.33	Current study
LIAISON XL FGF23 (CLIA)	France	2017	18–89	908	Mean ± 2SD	22.7–93.1	Souberbielle et al. (12)
Immunotopics FGF23 (ELISA)	Australia	2012	Adults (mean 56)	170	Nonparametric (2.5th–97.5th)	11.7–48.6	Smith et al. (15)
Kainos FGF23 (ELISA)	Japan	2002	21–63	104	Range	8.2–54.3	Yamazaki et al. (5)
Determinar CL FGF23 (CLEIA)	Japan	2022	Adults (median 53)	380	Mean ± 2SD	16.1–49.3	Kato et al. (6)
Medfrontier intact FGF23 (CLEIA)	Japan	2023	Adults (median 54)	380	Mean ± 2SD	18.6–59.8	Kato et al. (7)

SD, standard deviation; CLIA, chemiluminescent immunoassay; ELISA, enzyme-linked immunosorbent assay; CLEIA, chemiluminescent enzyme immunoassay.

Notably, intervals from both our study and that of Souberbielle et al. substantially exceed those reported in earlier investigations using different assay platforms: Smith et al. (11.7–48.6 pg/mL) (15), Yamazaki et al. (8.2–54.3 pg/mL) (5), and recent Japanese studies by Kato et al. (6, 7) (**Table 4**). This systematic divergence underscores well-documented analytical and calibration heterogeneity among measurement procedures employing distinct antibody clones, calibrator matrices, and signal transduction chemistries (1, 4, 6, 12–14). The magnitude of between-method bias reinforces the importance of method-specific RIs. Until metrological harmonization is achieved, population- and method-specific intervals are indispensable for accurate clinical interpretation.

Recently, Coşkun et al. proposed a biological variation (BV)-based approach to population RI estimation (22), in which relatively few reference individuals, as few as 30, suffice to determine the population set point (PSP), irrespective of data distribution. This PSP is then combined with within-subject BV (CV_I_), between-subject BV (CV_G_), and analytical variation (CV_A_) to derive a theoretical RI. According to the EFLM Biological Variation Database, CV_I_ and CV_G_ for FGF23 are 12.4% and 34.0%, respectively (23). Applying the log-transformed calculation method described by Coşkun et al., with our assay’s CV_A_ of 6.7% and the PSP derived from our cohort, we obtained a BV-based theoretical RI of 35.39-102.40 pg/mL (R code provided in **Supplementary Figure 3**), which shows close agreement with our conventional nonparametric RI of 28.04-100.33 pg/mL. Because identical BV estimates can be applied across any measurement system, this approach offers a practical and resource-efficient strategy for establishing method-specific RIs, particularly in the current pre-harmonization era.

A cardinal observation was the pronounced age dependence of iFGF23, with substantially lower concentrations in young adults aged 20–29 years. Harris–Boyd analysis conclusively supported age stratification, with z-statistics exceeding critical thresholds (z = 4.29–6.28, all z > z*), justifying distinct RIs: 25.73–78.76 pg/mL for young adults versus 32.01–107.00 pg/mL for adults ≥30 years (**Table 2**). Supplementary sex-stratified analysis confirmed that this age effect persisted independently in both males and females, with no significant differences among older age groups within either sex (**Supplementary Figure 2**), validating our dichotomous partitioning approach. This requirement for age-related partitioning has not emerged in French (12), Australian (15) or Japanese (5–7) cohorts (**Table 4**), yet aligns with studies documenting lower upper reference limits in children (24–26) (**Supplementary Table 3**), suggesting a population-specific maturation pattern.

From a mechanistic perspective, the age-related iFGF23 increase occurred in parallel with multiple established regulators of FGF23 transcription and metabolism: older adults exhibited significantly higher PTH (33.1 vs. 28.7 pg/mL, *p* = 0.002), higher 25-hydroxyvitamin D (30.5 vs. 26.2 ng/mL, *p* < 0.001), lower eGFR (92 vs. 115 mL/min/1.73 m^2^, *p* < 0.001), and higher alkaline phosphatase (70 vs. 61 IU/L, *p* < 0.001), yet paradoxically lower serum phosphate (3.6 vs. 3.9 mg/dL, *p* < 0.001) (**Table 3**). However, despite the robust between-group differences in multiple regulators, individual-level correlations between iFGF23 and traditional mineral metabolism parameters (phosphate, calcium, PTH, 25-hydroxyvitamin D) were notably absent (all |*r*| < 0.10, *p* > 0.05; **Supplementary Table 2**, **Supplementary Figure 1**), whereas correlations with age, eGFR, and alkaline phosphatase were significant. This pattern suggests that age-related FGF23 variation reflects coordinated, multifactorial influences rather than the isolated effects of individual calciotropic hormones or mineral ions. Concurrent changes in multiple physiological variables in older adults likely exert synergistic effects on FGF23 expression, which were not captured by univariate analysis of individual mineral metabolism parameters.

Males exhibited significantly elevated median iFGF23 (65.03 vs. 51.98 pg/mL, *p* < 0.001); however, Harris–Boyd analysis did not support sex-based partitioning (z = 5.17, z* = 5.38). While central tendencies differ, the Harris–Boyd method evaluates whether separate RIs would meaningfully reduce misclassification at distributional tails, the clinically decisive regions for detecting pathological elevation or suppression (20, 27). The marginal partitioning test results suggests that the practical clinical utility of sex-specific intervals warrants empirical evaluation in prospective studies.

## 5 Strengths and limitations

This investigation is strengthened by its large, rigorously defined reference population (*n* = 386), strict adherence to CLSI EP28-A3c guidelines, comprehensive statistical methodology including multiple normality assessments and transformation strategies, systematic partitioning analysis using established Harris–Boyd criteria, and the use of a fully automated, widely deployed *in vitro* diagnostic platform ensuring direct clinical applicability. Nevertheless, several limitations warrant acknowledgment.

First, as this was a single-center study in a Korean population, generalizability to other ethnic groups may be constrained, particularly given the observed age-partitioning requirement absent in other cohorts. Second, data on potential confounders, including dietary phosphate intake, iron status as a known transcriptional regulator of FGF23 (28), and soluble klotho concentration (29, 30), were unavailable. Third, phlebotomy was not time-standardized. Given the documented diurnal variation in FGF23 secretion (5), this introduced pre-analytical variability that may have widened the derived RIs. Fourth, the 30–39 year age group comprised only 12 participants, which may have limited the statistical power to detect subtle differences within the older age strata, although this did not affect our primary age partitioning decision. Fifth, establishing a clinical decision limit for this assay was beyond the scope of this study. A cutoff of 30 pg/mL, validated using the Kainos enzymatic immunoassay (5), is widely used to differentiate FGF23rHR from other etiologies of hypophosphatemia. However, owing to the significant inter-assay variability, this threshold cannot be directly applied to the LIAISON XL platform. A formal diagnostic accuracy study is warranted to determine the optimal clinical decision limit for this assay to differentiate patients with FGF23rHR from those with other causes of phosphate dysregulation.

## 6 Conclusions

This study provides robust, method-specific RIs for intact FGF23 levels in healthy Korean adults, with age-stratified benchmarks essential for clinical interpretation. Sex-based partitioning was not warranted, despite the higher median concentrations in males. These findings underscore the need for population- and method-specific reference data in the absence of assay harmonization. The observed age-dependent pattern, which aligns with lower pediatric values, warrants prospective longitudinal studies tracking FGF23 from childhood through adulthood using a consistent methodology, coupled with mechanistic investigations to determine whether these findings reflect population-specific physiology or methodological and environmental factors.

## Data Availability

The raw data supporting the conclusions of this article will be made available by the authors, without undue reservation.
