# Supplementary material for: Reference intervals for intact FGF 23 in healthy Korean adults: lower concentrations in young adulthood require age-specific partitioning
Source: Front Endocrinol (Lausanne). 2026 Mar 6;17:1730871. doi: 10.3389/fendo.2026.1730871 (PMC13002424; doi:10.3389/fendo.2026.1730871)
Supplement: Supplementary file 1 [file Supplementaryfile1.pdf]

Table S1. Baseline characteristics of the reference population

	Total (n = 386)		Male (n = 193)		Female (n = 193)		p-value[a]
Age, years	53	[28–63]	52	[29–62]	53	[27–64]	0.570
BMI, kg/m^2	23.4	[21.7–25.6]	24.7	[23.0–26.1]	22.1	[21.2–23.7]	< 0.001
SMM, kg	25.3	[20.6–30.3]	29.8	[27.5–32.6]	20.5	[18.7–21.9]	< 0.001
BFM, kg	17.8	[15.2–21.8]	17.9	[15.0–22.1]	17.8	[15.9–21.3]	0.904
eGFR, mL/min/1.73 m^2	96	[87–107]	94	[84–102]	99	[91–113]	< 0.001
iFGF23, pg/mL	58.26	[46.21–71.77]	65.03	[51.39–77.74]	51.98	[42.00–65.70]	< 0.001
Phosphate, mg/dL	3.7	[3.3–4.0]	3.5	[3.2–3.8]	3.9	[3.6–4.2]	< 0.001
Calcium, mg/dL	9.6	[9.2–9.8]	9.6	[9.3–9.8]	9.5	[9.2–9.8]	0.003
PTH, pg/mL	32.1	[25.2–40.4]	32.9	[25.2–41.6]	31.3	[25.3–38.8]	0.186
25(OH)D, ng/mL	28.7	[23.6–36.0]	27.7	[23.2–34.1]	30.0	[23.9–37.9]	0.059
ALP, IU/L	68	[58–79]	63	[56–76]	67	[58–79]	< 0.001

Data presented as median [interquartile range].

[a]Group differences between males and females were tested with the Mann–Whitney *U* test.

Abbreviations: BMI, body mass index; SMM, skeletal muscle mass; BFM, body fat mass; eGFR, estimated glomerular filtration rate; iFGF23, intact fibroblast growth factor 23; PTH, parathyroid hormone; 25(OH)D, 25-hydroxyvitamin D; ALP, alkaline phosphatase.

Table S2. Pearson correlation between intact FGF23 and other clinical parameters

	r	*p*-value
Age, years	0.278	< 0.001
eGFR, mL/min/1.73 m^2	-0.254	< 0.001
Phosphate, mg/dL	-0.061	0.235
Calcium, mg/dL	0.087	0.086
PTH, pg/mL	0.018	0.731
25(OH)D, ng/mL	0.093	0.068
ALP, IU/L	0.143	0.005

Abbreviations: eGFR, estimated glomerular filtration rate; PTH, parathyroid hormone; 25(OH)D, 25-hydroxyvitamin D; ALP, alkaline phosphatase

Table S3. Comparison of reference intervals for intact FGF23 in pediatric populations

Assay (platform)	Country	Year	Age range (years)	*n*	Statistical method	Reference interval (pg/mL)	Reference
LIAISON XL FGF23 (CLIA)	Poland	2021	<1	19	99th percentile	≤68.39	Stęńczyk et al. (24)
			1–4	28	99th percentile	≤56.74	
			5–9	32	99th percentile	≤53.96	
			10–14	27	99th percentile	≤32.55	
			15–18	15	99th percentile	≤48.08	
LIAISON XL FGF23 (CLIA)	Italy	2022	1–18	115	Upper reference limit	≤61.21	Brescia et al. (23)
LIAISON XL FGF23 (CLIA)	Italy	2024	Infants	30	Range	11.2–90.3	Baroncelli et al. (22)
			Prepubertal	147	Range	16.5–75.8	
			Pubertal	59	Range	19.8–91.1	
			Postpubertal	46	Range	15.6–87.3	

Abbreviations: CLIA, chemiluminescent immunoassay.

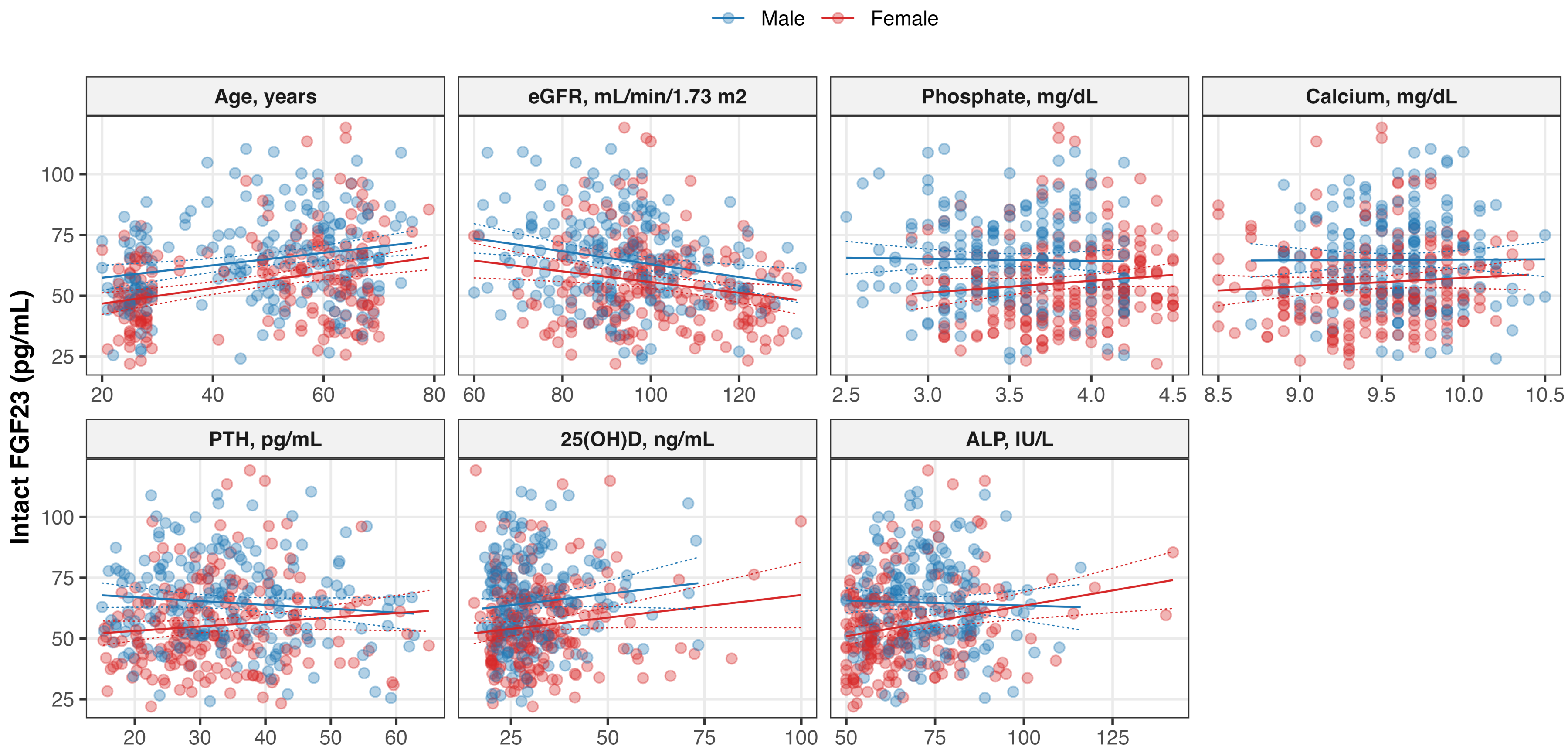

Figure S1. Correlations of intact FGF23 concentrations with demographic and biochemical parameters

Scatter plots of intact FGF23 concentrations versus clinical parameters stratified by sex: males (blue), females (red) (n = 193 each). Solid lines represent linear regression fits with 95% confidence intervals (dotted lines).

Abbreviations: eGFR, estimated glomerular filtration rate; PTH, parathyroid hormone; 25(OH)D, 25-hydroxyvitamin D; ALP, alkaline phosphatase.

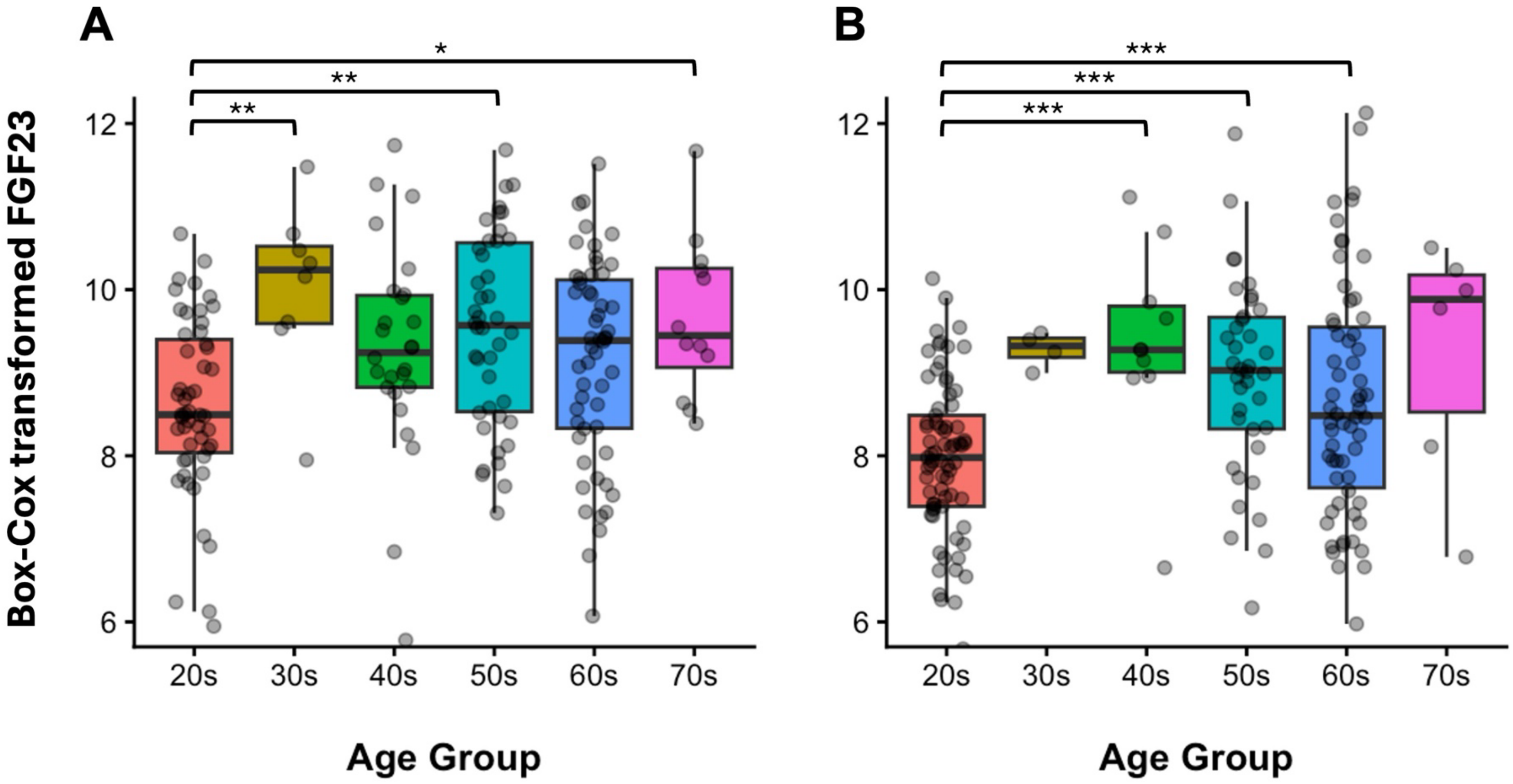

Figure S2. Distribution of intact FGF23 concentrations across age groups by sex

Dot plots and boxplots of Box–Cox transformed (λ = 0.343) intact FGF23 values stratified by age group for (A) males and (B) females. Statistical significance determined by one-way ANOVA with Tukey's HSD post-hoc test: *$p < 0.05$, **$p < 0.01$, ***$p < 0.001$

```r
# Input parameters
n <- 386; CVI <- 0.14; CVG <- 0.225; CVA <- 0.067

# Step 1: Estimate PSP from log-normal data (Equation 5)
mu <- mean(log(ref_data$FGF23), na.rm = TRUE)
sigma <- sd(log(ref_data$FGF23), na.rm = TRUE)
PSP <- exp(mu + 0.5 * sigma^2)

# Step 2: Convert CV estimates to log-scale SD
SD_I <- sqrt(log(1 + CVI^2))
SD_G <- sqrt(log(1 + CVG^2))
SD_A <- sqrt(log(1 + CVA^2))

# Step 3: Calculate total variation (Equation 2, log-scale)
SD_star <- sqrt((n + 1) / n * (SD_I^2 + SD_G^2 + SD_A^2))

# Step 4: Derive BV-based popRI limits (Equation 6)
RI_lower <- exp(mu + 0.5 * sigma^2 - 1.96 * SD_star)
RI_upper <- exp(mu + 0.5 * sigma^2 + 1.96 * SD_star)
```

Figure S3. R code for BV-based reference interval calculation

Computational algorithm for deriving biological variation (BV)-based reference intervals. The provided R script demonstrates the integration of empirical study data with established BV components (CV_I and CV_G) from the EFLM database and local analytical performance (CV_A). The model adopted herein assumes a log-normal distribution to estimate the population set point (PSP) and the total theoretical dispersion (SD*), as described by Coşkun et al. (22). Variables in red denote study-specific parameters and should be modified accordingly when applied to other datasets.
